# Pentosan polysulfate promotes proliferation and chondrogenic differentiation of adult human bone marrow-derived mesenchymal precursor cells

**DOI:** 10.1186/ar2935

**Published:** 2010-02-18

**Authors:** Peter Ghosh, Jiehua Wu, Susan Shimmon, Andrew CW Zannettino, Stan Gronthos, Silviu Itescu

**Affiliations:** 1 Proteobioactives Pty Ltd, 27/9 Powells Road, Brookvale, New South Wales 2100, Australia; 2 Mesoblast Ltd, Level 39, 55 Collins Street, Melbourne, Victoria 3000, Australia; 3 Myeloma Research Laboratory, Centre for Cancer Biology, Division of Haematology, Hansen Institute and Institute of Medical and Veterinary Science, Frome Road, Adelaide, South Australia 5000, Australia; 4 Mesenchymal Stem Cell and Regenerative Medicine Group, Bone and Cancer Laboratories, Division of Haematology, Hansen Institute and Institute of Medical and Veterinary Science, Frome Road, Adelaide, South Australia 5000, Australia

## Abstract

**Introduction:**

This study was undertaken to determine whether the anti-osteoarthritis drug pentosan polysulfate (PPS) influenced mesenchymal precursor cell (MPC) proliferation and differentiation.

**Methods:**

Human MPCs were maintained in monolayer, pellet or micromass cultures (MMC) for up to 10 days with PPS at concentrations of 0 to 20 μg/ml. MPC viability and proliferation was assessed using the WST-1 assay and ^3^H-thymidine incorporation into DNA, while apoptosis was monitored by flow cytometry. Proteoglycan (PG) biosynthesis was determined by ^35^SO_4_^2- ^incorporation and staining with Alcian blue. Proteoglycan and collagen type I and collagen type II deposition in pellet cultures was also examined by Toluidine blue and immunohistochemical staining, respectively. The production of hyaluronan (HA) by MPCs in MMC was assessed by ELISA. The relative outcome of PPS, HA, heparin or dextran sulfate (DS) on PG synthesis was compared in 5-day MMC. Gene expression of MPCs in 7-day and 10-day MMC was examined using real-time PCR. MPC differentiation was investigated by co-culturing with PPS in osteogenic or adipogenic inductive culture media for 28 days.

**Results:**

Significant MPC proliferation was evident by day 3 at PPS concentrations of 1 to 5 μg/ml (*P *< 0.01). In the presence of 1 to 10 μg/ml PPS, a 38% reduction in IL-4/IFNγ-induced MPC apoptosis was observed. In 5-day MMC, 130% stimulation of PG synthesis occurred at 2.5 μg/ml PPS (*P *< 0.0001), while 5.0 μg/ml PPS achieved maximal stimulation in the 7-day and 10-day cultures (*P *< 0.05). HA and DS at ≥ 5 μg/ml inhibited PG synthesis (*P *< 0.05) in 5-day MMC. Collagen type II deposition by MMC was significant at ≥ 0.5 μg/ml PPS (*P *< 0.001 to 0.05). In MPC-PPS pellet cultures, more PG, collagen type II but less collagen type I was deposited than in controls. Real-time PCR results were consistent with the protein data. At 5 and 10 μg/ml PPS, MPC osteogenic differentiation was suppressed (*P *< 0.01).

**Conclusions:**

This is the first study to demonstrate that PPS promotes MPC proliferation and chondrogenesis, offering new strategies for cartilage regeneration and repair in osteoarthritic joints.

## Introduction

Adult mesenchymal stem cells (MSCs) are an abundant source of self-renewing, multipotent undifferentiated cells that can be readily isolated from bone marrow, adipose tissue, muscle and synovium, and can be expanded in *ex vivo *culture [1,2]. The ability of these cells to differentiate into bone, cartilage, adipose, tendon and other cells of the mesenchymal lineage under appropriate stimuli offers the potential for the regeneration and repair of the musculoskeletal system by their direct application to sites of injury [3-5] or by their incorporation into bioscaffolds and transplantation into the sites of the tissue defect [3,6,7].

Stromal tissue in the bone marrow consists of a heterogeneous population of MSCs that occupy a perivascular niche [8-10]. Recent studies, however, provide evidence for the existence within this niche of smaller precursor stem cell populations that exhibit extensive proliferative and multilineage differentiative capacity and can be distinguished by their expression of certain cell surface antigens [11,12]. These undifferentiated mesenchymal precursor cells (MPCs) can be isolated from bone marrow aspirates using magnetic activated cell sorting in combination with antibodies that identify STRO-1, VCAM-1 (CD106), STRO-3 (tissue nonspecific alkaline phosphatase), STRO-4 (HSP-90b) and CD146 [11,12]. Using this approach, a homogeneous population of quiescent MPCs can be obtained that lack the phenotypic characteristics of leukocytes and mature stromal elements and exhibit extensive proliferative capacity while retaining the ability to differentiate into bone, cartilage and adipose tissues. This technique of selecting MPCs from bone marrow aspirates therefore provides a purer population of uncommitted bone marrow MSCs than can be obtained using traditional methods of isolation that rely on ficol gradient centrifugation, followed by plastic adherence in culture flasks [1,2]. Subcutaneous implantation of STRO-1^+^, STRO-3^+^, STRO-4^+^, CD106^+ ^and CD146^+ ^MPCs together with an osteoinductive hydroxy apatite/tricalcium phosphate carrier into NOD/SCID mice resulted in the successful deposition of bone at the site of implantation [11-13].

Parallel experiments designed to generate cartilage *in vivo *using the same model, however, necessitated subcutaneous implantation of MPCs combined with transforming growth factor (TGF)-β3 within fibrin glue (A.C.W. Zannettino and S. Gronthos, unpublished observations). Even though the TGF-β/bone morphogenetic protein (BMP) superfamily of growth factors are the most extensively used inducers of MSC chondrogenic differentiation [3-7,14,15], the co-administration of these proteins with MSCs directly into tissues could be problematic. For example, these proteins generally have short half-lives *in vivo *[14], and injection of TGF-β3 or BMP-2 directly into synovial joints of animal models has been reported to promote fibrosis and osteophyte formation [16,17]. These reports highlight a need to identify alternatives to the established growth factors (such as the TGF-β/BMP superfamily) that could be co-administered with MPCs and promote chondrogenic differentiation without the potential of adverse side effects, or the need to include slow release carriers/scaffolds to extend their time of exposure to the target cells.

Pentosan polysulfate (PPS) is a semi-synthetic sulfated polysaccharide that has been used for a number of therapeutic applications, including osteoarthritis [18]. Moreover, extensive *in vitro *and animal model studies using the sodium or calcium salts of PPS have shown that these molecules are effective at reducing joint inflammation, promoting fibrinolysis, stimulating hyaluronan (HA) synthesis by synovial fibroblasts and stimulating proteoglycan (PG) synthesis by chondrocytes [18-20]. The PPS salts are also potent inhibitors of granulocyte elastase and the serine proteinases of the complement and fibrinolytic systems, as well as downregulating MMP-13 production at the gene promotor level ([18-21] and references cited within). Recent studies have also identified the inhibitory effects of PPS on the cartilage aggrecanases, ADAMTS4 and ADAMTS5 [22,23], and their binding to the endogenous inhibitor of these matrix metalloproteinases, tissue inhibitor of metalloproteinases-3, as well as increasing its extracellular half-life [23,24]. PPS thus possesses the ability to stimulate the biosynthesis of components of the extracellular matrix while concomitantly limiting their degradation by its direct and indirect anti-catabolic effects. As already mentioned, PPS has been used for the treatment of osteoarthritis in both veterinary [18,25,26] and human [18,27] practice, where it is administered via the oral, systemic or intraarticular routes to provide pain relief and restoration of joint function.

The present study was undertaken to determine whether PPS was capable of promoting chondrogenic differentiation of MPCs *in vitro *as a first step in the development of an improved modality for restoring cartilage integrity in degenerative synovial joints and the intervertebral disc. The outcome of these *in vitro *experiments confirmed that PPS possessed the ability to induce proliferation and chondrogenic differentiation of MPCs while inhibiting osteogenic differentiation.

## Materials and methods

### Drugs

Sodium PPS (batch# Q18) was supplied by bene-Arzneimittel GmbH (Munich, Germany), HA (SupaArtz^®^) was obtained from Seikagaku Kogyo Company (Tokyo, Japan), and dextran polysulfate sulfate (molecular weight = 5,000 Da) and heparin were purchased from Sigma-Aldrich (Sydney, Australia). The sources of all other chemicals and reagents are identified in the text.

### Mesenchymal progenitor cells

STRO-3-positive, human bone marrow-derived MPCs (Batch# M111sP4) were prepared by Lonza (Walkersville, MD, USA) for Mesoblast Ltd (Melbourne, Australia), according to the isolation procedure originally described by Gronthos and colleagues [9,13]. The same batch of MPCs was used for all of the culture experiments described in this study.

### MPC viability and proliferation using the mitochondrial dehydrogenase cleavage assay

The method used has been described previously [9]. Briefly, primary human MPCs (8 × 10^3 ^cells/well) were cultured in 24-well plates in growth media supplemented with 0, 1, 2, 5 and 10 μg/ml PPS in quadruplicate cultures. On days 1, 3 and 6, the growth media was removed and replaced with phenol-red-free media containing the tetrazolium salt, WST-1, and was then incubated for 2 hours at 37°C in 5% CO_2_. The intensity of colour formation was detected using an ELISA microtitre plate reader at a wavelength of 450 nm.

### Biosynthesis of DNA by MPCs in monolayer cultures

MPCs (100 μl) (2 × 10^4 ^cells/ml, 2,000 cells/well) were seeded into wells of a 96-well culture plate and incubated with 200 μl DMEM-high glucose medium containing 10% FBS at 37°C in a humidified atmosphere of 5% CO_2 _for 24 hours. The medium was then replaced with 200 μl DMEM-high glucose (+10% FBS) containing PPS and ^3^H-thymidine (Perkin-Elmer Life and Analytical Science, Knoxfield, Victoria, Australia) to afford final well concentrations of 0, 0.1, 0.5, 1.0, 2.5, 5.0, 10.0 and 20.0 μg/ml and 5 μCi/ml, respectively. Quadruplicate cultures of each PPS concentration were used. Cultures were incubated at 37°C in humidified 5% CO_2 _for 72 hours. The medium was then removed and the wells were rinsed twice with 200 μl PBS followed by addition of 50 μl PBS containing 0.25% trypsin (Sigma-Aldrich). The cultures were incubated at 37°C for 10 minutes and the released cells were lysed by addition of 200 μl dH_2_O to each well for 20 minutes at room temperature. The ^3^H-DNA in the cell lysates was separated from unincorporated ^3^H-thymidine using glass fibre filters and a cell harvester (Skatron Instruments AS, Lier, Norway). The filters were then air-dried and punched into scintillation vials. Scintillation cocktail liquid (3 ml/vial) was added and vials were vortexed for 30 to 40 seconds. The radioactivity of ^3^H incorporated into the DNA of replicating MPCs was determined by scintillation counting (Tricarb 2900 TR; Perkin-Elmer Life and Analytical Science).

### Pentosan polysulfate and MPC apoptosis

The method used has been described previously [28]. Briefly, single-cell suspensions of cultured MPCs (2 × 10^4 ^cells/well) were plated in serum-free media supplemented with 0, 1, 2, 5 and 10 μg/ml PPS. MPC apoptosis was induced by the addition of a combination of 30 ng/ml IL-4 and 30,000 U/ml IFNγ/well. Following 5 days of culture, cells were harvested by trypsinisation and MPC viabilities were assessed by Annexin V staining as previously described [28].

### Concentration effects of PPS on the biosynthesis of proteoglycans by MPCs in micromass cultures over 5, 7 and 10 days

MPCs were established in micromass cultures (MMC) using the methodology described previously [29-31]. Ten microlitres of a single-cell suspension of MPCs (1 × 10^7 ^cells/ml) in DMEM-high glucose + 10% FBS were applied to the centre of individual wells of 24-well culture plates (Cellstar^®^; Greiner Bio-One GmbH, Frickenhausen, Germany). After incubation at 37°C in 5% CO_2 _for 3 hours, 900 μl DMEM-high glucose + 10% FBS was added and plates were then incubated for a further 20 hours. The media was replaced with DMEM-high glucose + 10% FBS containing PPS at final well concentrations of 0.0, 0.1, 0.5, 1.0, 2.5, 5.0, 10.0 and 20.0 μg/ml, and cultures were maintained with media changes containing the same PPS concentrations every 48 hours.

Two days prior to culture termination on days 3, 5, and 7, the media was replaced with media containing ^35^S-H_2_SO_4 _(Perkin-Elmer Life and Analytical Science) at a final ^35^SO_4 _well concentration of 5 μCi/ml together with the same concentrations of PPS and culture continued for a further 2 days. On days 5, 7 and 10 media were collected and cells released with 2.5% trypsin/ethylenediamine tetraacetic acid and collected by centrifugation. After washing with PBS, the cell pellets were stored at -70°C for RNA and DNA analysis. The supernatants and washings were combined and digested with Papain (Sigma-Aldrich) (2.5 mg/ml in 0.1 M Na acetate, 5 mM ethylenediamine tetraacetic acid, pH 6.0) at 65°C for 2 hours.

Aliquots (n = 6) of the digest were transferred to 96-well plates and the ^35^S-sulfated glycosaminoglycans (^35^S-GAGs) were separated from the ^35^SO_4 _by addition of 20 μl of 5% cetyl pyridinium chloride + 10 μl of 1 mg/ml chondroitin sulfate C (both from Sigma Chemicals, Sydney, Australia). The precipitate ^35^S-GAG-cetyl pyridinium chloride complexes were collected by filtration through glass fibre discs using a cell harvester (Skatron Instruments AS). The radioactivity of the ^35^S-GAG-cetyl pyridinium chloride complexes was determined by scintillation counting (Tricarb 2900 TR; Perkin-Elmer Life and Analytical Science). The incorporation of ^35^S into newly synthesised GAGs was normalised to the culture DNA content [32]. Results were calculated as ^35^S-GAG-Decays Per Minute/μg DNA and then expressed as a percentage of control cultures (without PPS) that were set at 100%.

### Concentration effects of PPS, hyaluronan, dextran sulfate and heparin on the biosynthesis of proteoglycans by MPCs in MMC over 5 days

The MMC, established as described above, were maintained in culture for 3 days in the presence of PPS, HA, dextran sulfate (DS) or heparin at concentrations of 0.0, 0.1, 0.5, 1.0, 2.5, 5.0, 10.0 and 20.0 μg/ml, and were then incubated with ^35^S-H_2_SO_4 _(Perkin-Elmer Life and Analytical Science) at a final ^35^SO_4 _well concentration of 5 μCi/ml for an additional 2 days in the presence of the drugs. Incorporation of ^35^S into S-GAGs was determined as described above and the data expressed as the percentage of controls cultured in the absence of the drugs.

### Concentration effects of PPS on proteoglycan production by MPCs in MMC over 5, 7 and 10 days as assessed by staining with Alcian blue

Micromass cultures of MPCs (10^7 ^cells/ml, 8 μl/micromass) were established on eight-well slides (Lab-Tek^® ^Chamber Slide, Permanox^®^; Grand Island, NY, USA) and maintained in the presence of 0 to 20 μg/ml PPS for 5, 7 and 10 days as described above for the PG synthesis assays. At the end of each culture period, the medium was removed and the cells and matrix were fixed with HistoChoice MB (Amresco, Solon, OH, USA). After washing in distilled water for 1 minute, the cultures were stained with 1% Alcian blue, 8GX (Sigma Chemical Company, Sydney, Australia) (dissolved in 3% acetic acid at pH 2.5) for 30 minutes. Staining was terminated by washing the slides with running tap water for 2 minutes, and then rinsing in distilled water for a further 2 minutes. After dehydrating through three changes of absolute ethanol, allowing 3 minutes for each change, the stained micromass cultures were observed microscopically and the images captured using a microscope digital camera (Nikon Eclipse 80i; Coherent Sciences, Adelaide, Australia).

### Concentration effects of PPS on collagen type II production by MPCs in MMC over 5, 7 and 10 days

MMC of MPCs were established in 24-well culture plates at a starting density of 8 × 10^4 ^cells/micromass in the presence of 0 to 20.0 μg/ml PPS as described above for the PG synthesis assays. On days 5, 7 and 10, media were removed and cultures were fixed with Histochoice MB (Amresco) for 20 to 30 minutes at room temperature and processed for immunohistochemical staining of collagen type II as described by Denker and colleagues [29]. A human collagen type II monoclonal antibody (Clone II-4II, IgG, ImmunO™; MP Biomedicals Australia, Seven Hills, NSW, Australia) dissolved in PBS/10% Hepes-Triton 10% FBS followed by a goat anti-mouse AP-conjugated secondary antibody (2 mg/ml) (Invitrogen Australia Pty Ltd, Mulgrave, Victoria, Australia) was used. After washing (3 × 5 minutes) with PBS, 200 μl 5-bromo-4-chloro-3'-indoyl phosphate/buffer + nitroblue tetrazolium salt was added and the cultures incubated at 37°C for 15 to 20 minutes. After the purple colour was fully developed (15 to 20 minutes) the wells were washed with running water that was discarded. The dried plates and wells were then photographed with a digital camera (Nikon D40×) and the digital images were analysed by Image J^® ^public domain software using a personal computer.

### Concentration effects of PPS on proteoglycan, collagen type I and collagen type II deposition by MPCs in 5-day, 7-day and 10-day pellet cultures

MPCs (3 × 10^5 ^cells) were seeded in sterile 2 ml screw-capped plastic centrifuge tubes (Scientific Specialities Inc., Lodi, CA, USA) to which 1 ml DMEM-high glucose medium + 10% FBS was added. Cells were centrifuged at 500 × *g *for 10 minutes at room temperature, the caps were loosened and the cultures maintained at 37°C in a 5% CO_2 _atmosphere to establish the pellets. After 24 hours the medium in each tube was removed and replaced with 1.0 ml DMEM-high glucose medium + 10% FBS containing PPS at final concentrations of 0.0, 0.1, 0.5, 1.0, 2.5, 5.0, 10.0 and 20.0 μg/ml, respectively. The pellet cultures were maintained under these conditions for 5, 7, and 10 days with medium changes with and without PPS every 2 to 3 days. Triplicate cultures were established for each of the PPS concentrations nominated. At the end of each culture period, the medium was aspirated and pellets were fixed by addition of 200 μl Histochoice MB (Amresco) for 45 minutes at room temperature, rinsed with 70% ethanol once and stored in 70% ethanol prior to paraffin embedding and processing for histological and immunohistochemical examination as described previously [9].

De-paraffinised 5 μm sections were stained with 0.1% Toluidine blue (Sigma Chemicals) or used for immunohistochemistry. Briefly, 5 μm serial sections were blocked for 2 hours with either 5% normal goat serum or 5% rabbit serum and were then incubated overnight at 4°C with the primary antibodies: goat anti-human immunoglobulin collagen type I (0.8 μg/ml; Chemicon International, Temecula, CA, USA), mouse anti-human IgG_1 _collagen type II (1:100; Chemicon International), or corresponding control antibodies goat immunoglobulin negative control (0.8 μg/ml; Caltag Laboratories, Burlingame, CA, USA) and mouse IgG_1 _(1B5) negative control (0.8 μg/ml, kindly provided by Prof. L. Ashmann, University of Newcastle, New South Wales, Australia). The sections were washed three times with PBS before adding the secondary antibodies of either conjugated goat anti-mouse IgG biotin (2.5 μg/ml; Southern Biotechnologies, Birmingham, AL, USA) or conjugated rabbit anti-goat immunoglobulin biotin (2.5 μg/ml; Vector Laboratories Inc. Burlingame, CA, USA), for 90 minutes at room temperature. After washing three times in PBS, the sections were incubated with streptavidin horseradish peroxidase (2 μg/ml; Chemicon International) for 1 hour at room temperature in a dark, moist chamber. The sections were then washed three times with PBS before incubating with the liquid DAB + Substrate Chromagen System according to the manufacturer's instructions (Dako, Carpinteria, CA, USA). After rinsing in distilled water, the sections were counterstained with Mayer's haematoxylin before mounting.

### Hyaluronan production by MPCs in MMC over 7 and 10 days

MPCs were established in MMC and cultured in the presence of 0.0, 0.1, 0.5, 1.0, 2.5, 5.0, 10.0 and 20.0 μg/ml PPS as described above. Experiments were terminated 7 and 10 days post seeding. Media were removed and centrifuged (12,000 × *g*/10 minutes), and triplicate aliquots were used to determine the HA concentrations using a commercially available ELISA kit (MP Biomedicals Asia Pacific Pte Ltd, Singapore) by following the manufacturer's instructions. To the remaining micromass culture in each well, 200 μl of 1 mg/ml collagenase (Sigma Chemicals) in collagenase buffer (66.7 mM NaCl, 6.7 mM KCl, 4.8 mM CaCl_2_, 10 mM HEPES (pH 7.4)) were added. The plates were incubated with gentle shaking at 37°C for 2.5 hours to release the cells. The cells and digested matrix were then placed in a boiling water bath for 30 minutes to denature the collagenase, cooled then centrifuged at 12,000 × *g *for 5 minutes. Triplicate aliquots of the supernatants were used to determine the HA concentrations within the matrices using the ELISA kit. Aliquots of the cell digests from each culture were used to determine the DNA content as described previously [32]. Results were expressed as micrograms per millilitre of HA per microgram of DNA to normalise for changing cell numbers.

### Real-time PCR

Real-time quantitative PCR was used to assess MPC expression of selected genes, as listed in Table 1, using the RT^2^Real-Time™ SYBR Green/Rox PCR master mix (SABiosciences Corporation, Frederick, MD, USA) according to the manufacturer's instructions. The typical Rotor-Gene (Corbett Research, Mortlake, NSW, Australia) cycling parameters used were: 50°C for 2 minutes; 95°C for 15 minutes (95°C for 15 seconds, 60°C for 26 seconds, 72°C for 10 seconds - 40 to 48 cycles); and 72°C for 3 minutes. The primer sets used for these studies are presented in Table 1. The cycle threshold values were used to calculate the relative mRNA copy number. All PCR reactions were performed in triplicate and validated by the presence of a single peak in the melt curve analysis. All expression was normalised against the housekeeping gene, β-actin.

**Table 1:** Primer sequences used for real-time PCR to evaluate gene expression by mesenchymal precursor cells

Gene	Primer sequences (5' to 3')
Aggrecan	Forward: CTG CTT CCG AGG CAT TTC
	Reverse: GCT CGG TGG TGA ACT CTA GG
Type I collagen	Forward: AGG GTC CCA ACG AGA TCG AGA TCC
	Reverse: TAC AGG AAG CAG ACA GGG CCA ACG TCG
Type II collagen	Forward: GCC TGG TGT CAT GGG TTT
	Reverse: GTC CCT TCT CAC CAG CTT TG
Sox-9	Forward: AGG TGC TCA AAG GCT ACG AC
	Reverse: GCT TCT CGC TCT CGT TCA GA
Type X collagen	Forward: AAT GCC CAC AGG CAT AAA AG
	Reverse: AGG ACT TCC GTA GCC TGG TT
IGFBP-3	Forward: CGC CAG GAA ATG CTA GTG AG
	Reverse: ACG GCA GGG ACC ATA TTC T
IGFBP-5	Forward: GAG AAA GCC CTC TCC ATG TG
	Reverse: TCA CGG GAG TCT CTC TCG AT
Noggin	Forward: ATC GAA CAC CCA GAC CCT ATC
	Reverse: TCT AGC CCT TTG ATC TCG CTC
CBFA-1 (RUNX2)	Forward: GTG GAC GAG GCA AGA GTT TCA
	Reverse: CAT CAA GCT TCT GTC TGT GCC
β-Actin	Forward: GAT CAT TGC TCC TCC TGA GC
	Reverse: GTC ATA GTC CGC CTA GAA GCA T

Primer sequences used for real-time PCR to evaluate gene expression by mesenchymal precursor cells maintained in micromass cultures in the absence and presence of pentosan polysulfate for 7 and 10 days. IGFBP, insulin-like growth factor binding protein.

### Concentration effects of PPS on MPC differentiation when cultured in an osteoinductive medium

The culture conditions required for the osteoblastic differentiation of MPCs and the deposition of mineralised bone matrix *in vitro *have been previously described [1,9]. The same osteoinductive medium was used for the present experiments and consisted of α-MEM supplemented with 2% (v/v) foetal calf serum, 1.8 mM KH_2_PO_4_, 10^-7 ^M dexamethasone, sodium phosphate, 50 IU/ml penicillin, 50 μg/ml streptomycin, 1 mM sodium pyruvate, 100 mM L-ascorbic acid 2-phosphate, 2 mM L-glutamine and 10 mM HEPES buffer. MPCs were seeded into 96-well plates at 8 × 10^3 ^cells/well and allowed to reach ≥ 90% confluence prior to the addition of osteoinductive media containing PPS at concentrations of 0.0, 1.0, 2.0, 5.0 and 10 μg/ml. Cells were cultured at 37°C in the presence of a humidified atmosphere of 5% CO_2 _with twice-weekly media changes for 4 weeks. The total mineral content per well was assessed from the calcium levels per DNA in each well [26]. Absolute absorbance was determined by measuring against a series of DNA standards at 350 nm in a LS55 Luminescence Spectrometer (Perkin Elmer Life and Analytical Science).

### Concentration effects of PPS on MPC differentiation when cultured in an adipogenic medium

MPCs were cultured in adipogenic-inductive media [27,33] comprised of complete α-MEM supplemented with 0.5 mM 3-isobutyl-1-methyl-xanthine, 60 μM indomethacin, and 0.5 μM hydrocortisone in the presence of concentrations of PPS of 0.0, 1.0, 2.0, 5.0 and 10 μg/ml. The inductive media was changed twice weekly for a period of 4 weeks. Cells were stained for the presence of lipid using Oil Red O by gently rinsed in 1 × PBS (pH 7.4) to avoid disruption of the cell monolayer. Cells were fixed in phosphate-buffered formalin for 15 minutes at 21°C. The fixative was subsequently removed and lipid stained by addition of 100 μl freshly filtered Oil Red O (3 mg/ml; MP Biomedicals Australia, Seven Hills, NSW, Australia) for ≥ 2 hours at ambient temperature. Cells were rinsed three times with water and counterstained with Mayer's haematoxylin (Lillie's modification). Haematoxylin stains were aspirated and replaced by water, and any Oil Red O-positive adipocytes were examined under a light microscope and photographed with a DP20-56 Olympus camera.

### Statistical analysis of data

The statistical analysis of the data generated in all assays that compared the effects of drug groups at different concentrations on MPC response relative to control (no drugs) response was undertaken using one-way analysis of variance with a Bonferroni multiple comparison *post hoc *test and Student's unpaired *t *test. Groups were considered significantly different from controls when *P *< 0.05. Each data point represents the mean ± standard deviation of three to six samples unless otherwise indicated. Data analysis and graphical representations of results were undertaken using the Instat^® ^and Microsoft Excel^® ^software packages.

## Results

### MPC viability and proliferation in monolayer culture

**Figure 1:**
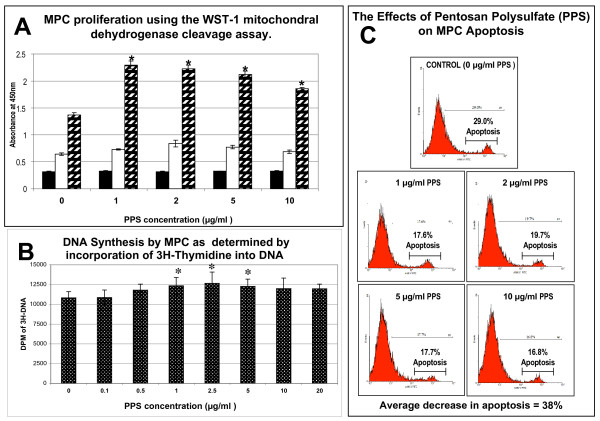
**Mesenchymal precursor stem cell viability and proliferation in monolayer culture**. **(a) **Bar graph showing the concentration-dependent effect of pentosan polysulfate (PPS) on mesenchymal precursor stem cell (MPC) proliferation. Primary MPCs were cultured in 24-well plates in growth media supplemented with PPS at the indicated concentrations (n = 3). On days 1 (black columns), 3 (white columns) and 6 (hatched columns), the cells were incubated with the tetrazolium salt WST-1 for 2 hours at 37°C to produce a formazan dye. Absorbance at 450 nm for each time point is shown for the indicated concentrations of PPS. Data expressed as mean ± standard error of the mean. A statistically significant increase in proliferation was observed on day 6 at concentrations of PPS in excess of 1 μg/ml (*P < 0.01). **(b) **Concentration effects of PPS on DNA synthesis in 3-day monolayer cultures of MPCs (n = 3). Data expressed as mean ± standard deviation. Significant elevation in DNA synthesis was observed at PPS concentrations of 1, 2.5 and 5 μg/ml relative to control cultures (*P *< 0.01). **(c) **Flow cytometric analysis profiles showing the inhibitory effects of different concentrations of PPS on MPC apoptosis induced by the addition of a combination of 30 ng/ml IL-4 plus 30,000 U/ml IFNγ. Following 5-day culture, cells were harvested by trypsinisation and viabilities assessed by Annexin V staining (n = 3 per PPS concentration). An average 38% reduction in apoptosis was observed when MPCs were cultured at PPS concentrations >1 μg/ml. DPM, Decays Per Minute.

Using the WST-1 mitochondrial dehydrogenase cleavage assay at day 6, viable MPC numbers were significantly increased in the cultures containing 1 to 10 μg/ml PPS (*P *< 0.01) (Figure 1a). Similarly, using ^3^H-hymidine incorporation, a significant increase in MPC numbers was observed at 1 to 5 μg/ml (*P *< 0.05) after 3 days of culture (Figure 1b). To determine whether the increase in MPC number was related to the anti-apoptotic properties of PPS, MPC monolayer cultures were exposed to IL-4 and IFNγ [31] in the absence and presence of the drug for 5 days. These experiments showed that PPS over the concentration range of 1 to 10 μg/ml diminished MPC apoptosis by an average of 38% relative to the control cultures containing no PPS (Figure 1c).

### Effects of PPS on proteoglycan synthesis in 5-day, 7-day and 10-day MPC MMC

**Figure 2:**
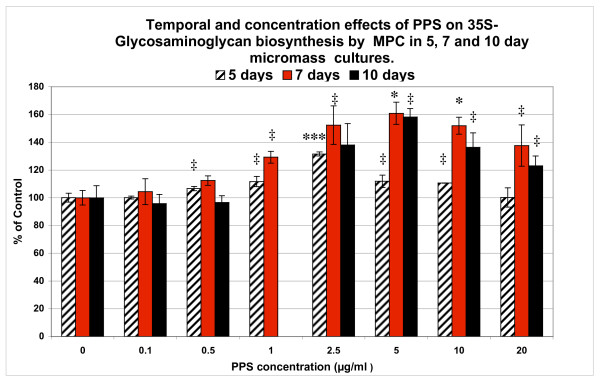
**Effects of pentosan polysulfate on proteoglycan synthesis in mesenchymal precursor stem cell micromass cultures**. Bar graphs showing the temporal and concentration effects of pentosan polysulfate (PPS) on the biosynthesis of proteoglycans (determined as sulfated glycosaminoglycans (^35^S-GAGs)) by mesenchymal precursor stem cells (MPCs) in micromass cultures for 5, 7 and 10 days. Data (mean ± standard deviation) presented as percentage of control calculated from the ^35^S-GAG DPM/μg DNA values at each PPS concentration (n = 6). ^‡^*P *< 0.05, *P < 0.01; **P < 0.001; ***P < 0.0001 relative to control cultures.

The incorporation of PPS in MPC MMC stimulated PG biosynthesis in a concentration-dependant manner that also varied with the duration of culture (Figure 2). In 5-day MMC, maximal stimulation (130%) of PG synthesis occurred at 2.5 μg/ml PPS (*P *< 0.0001), while 5.0 μg/ml PPS was required to achieve maximal stimulation (160%, *P *< 0.01) on day 7. Stimulation (150%, *P *< 0.05) was also observed, however, with the lower concentration of 2.5 μg/ml PPS in this time period. A similar concentration-dependent response to PPS as that for day 7 was found for the 10-day MPC cultures, with a maximal production of ^35^S-GAGs with 5.0 μg/ml PPS (*P *< 0.05) (Figure 2).

### Effects of PPS, hyaluronan, dextran sulfate and heparin on proteoglycan biosynthesis by MPCs

**Figure 3:**
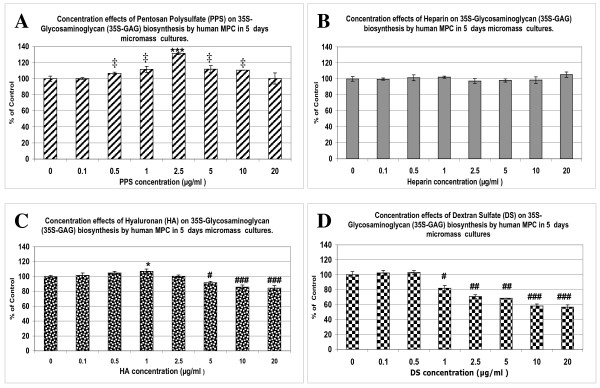
**Effects of pentosan polysulfate, hyaluronan, dextran sulfate and heparin on mesenchymal precursor cell proteoglycan biosynthesis**. Bar graphs showing the concentration-dependent effects of **(a) **pentosan polysulfate (PPS), **(b) **heparin, **(c) **hyaluronan (HA) and **(d) **dextran sulfate (DS) on prosteoglycan (PG) synthesis by mesenchymal precursor stem cells (MPCs) in micromass cultures for 5 days. Data (mean ± standard deviation) presented as percentage of control calculated from the sulfated glycosaminoglycan (^35^S-GAG) Decays Per Minute/μg DNA values at each drug concentration (n = 3). ^‡^*P *< 0.05, *P < 0.01; **P < 0.001; ***P < 0.0001 relative to control values.

As noted above, in the 5-day cultures (Figure 2) PPS exhibited a concentration-dependent stimulation of PG biosynthesis by MPCs, with a maximum effect being observed at 2.5 μg/ml PPS, with a progressive decline in stimulation at higher concentrations. Nevertheless, significantly higher levels of ^35^S-GAGs than controls were still produced by the MPCs at 5.0 and 10 μg/ml PPS (*P *< 0.05) (Figure 3a). Under the same culture conditions, heparin had no effect (Figure 3b) while HA stimulated PG synthesis at 1 μg/ml (*P *< 0.01) but significantly inhibited synthesis at concentrations of 5 to 20 μg/ml (*P *< 0.05 to 0.0005) (Figure 3c). DS was found to strongly inhibit PG synthesis over the concentration range of 1 to 20 μg/ml (*P *< 0.05 to 0.005) (Figure 3d).

### Effects of PPS on proteoglycan deposition by MPCs in MMC as assessed by Alcian blue staining

**Figure 4:**
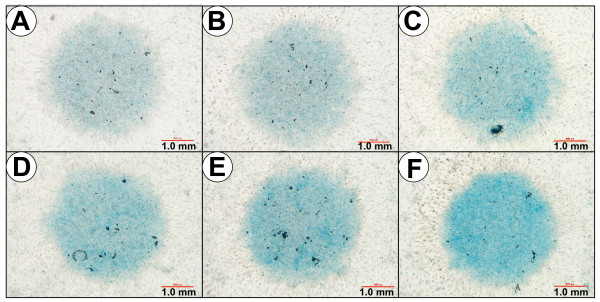
**Effects of pentosan polysulfate on proteoglycan deposition by mesenchymal precursor cells in micromass cultures**. Photomicrographs of mesenchymal precursor stem cell micromass cultures maintained in the absence and presence of pentosan polysulfate (PPS) for 10 days then stained with Alcian blue as described in the text: **(a) **0.0 μg/ml PPS, **(b) **1.0 μg/ml PPS, **(c) **2.5 μg/ml PPS, **(d) **5.0 μg/ml PPS, **(e) **10.0 μg/ml PPS, and **(f) **20 μg/ml PPS. Note the more intense staining with the dye at concentrations ≥ 2.5 μg/ml PPS, indicating the presence of higher levels of proteoglycans in these cultures.

Although the micromass cultures of MPCs established on the Nunc eight-well Lab-Tek^® ^Chamber Slides did not grow as well as on the Greiner Cellstar^® ^24-well plates, a gradation of staining for PGs with Alcian blue was found to be proportional to the incubation time and PPS concentrations used. The strongest staining was observed in the 10-day cultures, with the most intense Alcian blue staining evident at concentrations ≥ 2.5 μg/ml PPS (Figure 4).

### Effects of PPS on the deposition of collagen type II by MPCs in MMC as assessed by immunohistochemistry

**Figure 5:**
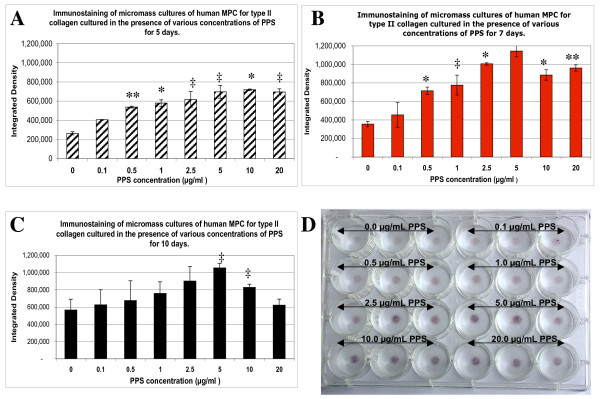
**Effects of pentosan polysulfate on collagen type II deposition by micromass culture mesenchymal precursor cells**. Bar graphs showing the concentration-dependent effects of pentosan polysulfate (PPS) on collagen type II deposition by mesenchymal precursor stem cells (MPCs) in micromass cultures (MMC) for **(a) **5 days, **(b) **7 days and **(c) **10 days as determined by digital analysis of colour-intensity images of the 5-bromo-4-chloro-3'-indoyl phosphate/buffer + nitroblue tetrazolium salt stained conjugate to collagen type II applied to fixed MMC. n = 3 for each PPS concentration. Data expressed as mean ± standard deviation. **(d) **Digital photograph of the stained 7-day MMC cultures in 24-well culture plates after processing as described in the text showing the staining intensity in relation to PPS concentration. ^‡^*P *< 0.05, *P < 0.01; **P < 0.001; ***P < 0.0001 relative to control.

Deposition of collagen type II in the fixed matrices of MPC MMC was successfully demonstrated and semi-quantified using the immunostaining technique described by Denker and colleagues [29]. For the 7-day and 10-day culture periods, a progressive stimulation of collagen type II synthesis was observed with PPS concentrations up to 5 μg/ml followed by a marked decline at 10 and 20 μg/ml PPS (Figure 5b, c, d). In the 5-day cultures, lower but significant (*P *< 0.001 to 0.05) stimulation of collagen type II synthesis by MPCs was still evident at PPS concentrations up to 20 μg/ml (Figure 5a).

### Effects of PPS on proteoglycan, collagen type I and collagen type II deposition by MPCs in 5-day, 7-day and 10-day pellet cultures

**Figure 6:**
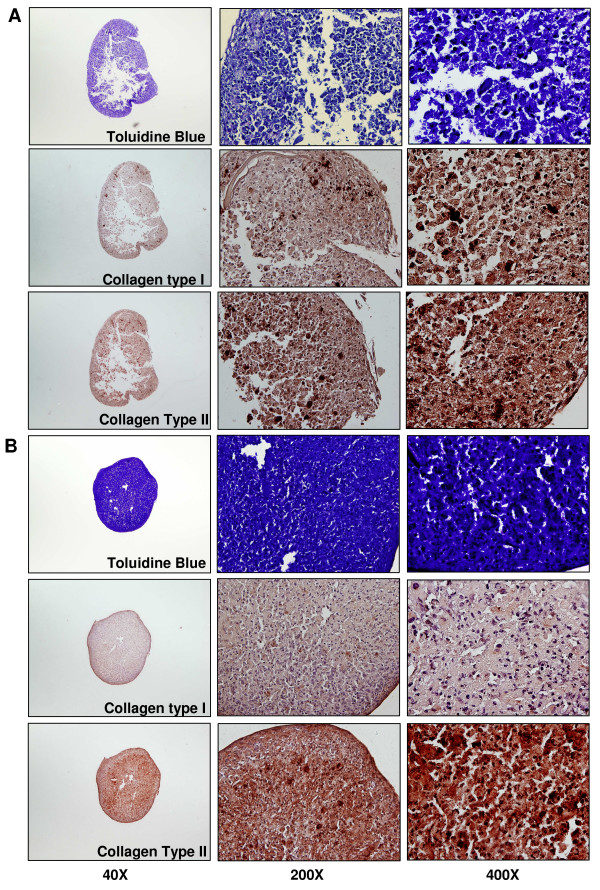
**Effects of pentosan polysulfate on proteoglycan, collagen type I and collagen type II deposition in pellet cultures**. Photomicrographs of sections of mesenchymal precursor stem cell (MPC) pellet cultures showing the effects of pentosan polysulfate (PPS) on chondrocyte development. Representative 5 μm serial tissue sections are shown of cells cultured as pellets for 10 days in **(a) **the absence of PPS (top nine panels) or **(b) **the presence of PPS (bottom nine panels). Control pellet sections (a) appeared fragmented with less intense toluidine blue staining for proteoglycans in comparison with those pellet cultures treated with 2.5 mg/ml PPS (b). Immunohistochemical staining confirmed higher levels of the cartilage matrix protein and collagen type II, and lesser levels of collagen type I in pellet cultures treated with 2.5 μg/ml PPS (b) than in the corresponding control pellet cultures (a).

Pellets were formed by all cultures irrespective of the time of incubation and the absence or presence of PPS. While no attempt was made to quantify the differences, larger more robust pellets were generally observed in the presence of PPS. Moreover, PPS also conferred a stronger and more uniform Toluidine blue staining for PGs, as exemplified in the photomicrographs of pellets shown in Figure 6a, b (top panels). Compact pellets and uniformity of staining in PPS co-cultures was also observed for matrix collagens in the serial sections used for immunohistochemical staining of these proteins. Moreover, the intensity of staining for collagen type II was greater than collagen type I in sections of pellets grown in the presence of 2.5 μg/ml PPS compared with control pellets cultured without PPS (compare Figure 6a with Figure 6b, bottom two panels).

### Effects of PPS on the synthesis of hyaluronan by MPCs

In the absence of PPS, MPCs were found to elaborate HA into the media and extracellular matrix in equal amounts in the 7-day MMC, but this distribution was altered in the presence of PPS with increasing amounts being deposited in the matrix (Table 2). This increase was significant at 5 and 10 μg/ml PPS (*P *< 0.001) while media levels declined accordingly (*P *< 0.05) (Table 2). In the 10-day MPC control cultures, higher levels of HA were detected in the matrix than in the culture media (*P *< 0.05). In the presence of all concentrations of PPS, HA levels in the media at 10 days were less than control media levels (*P *< 0.05) (Table 2). Matrix deposition of HA, however, was found to be higher than controls at PPS over the concentration range of 1.0 to 20 μg/ml but was only significant at 0.1 and 0.5 μg/ml PPS (*P *< 0.001) (Table 2).

**Table 2:** Concentration effects of pentosan polysulfate on hyaluronan deposition in human mesenchymal progenitor cell micromass cultures

PPS concentration (μg/ml)	HA concentration (μg/ml)			
	
	Media 7 days	Matrix 7 days	Media 10 days	Matrix 10 days
Control 0.0	1.54 ± 0.03	1.58 ± 0.01	1.55 ± 0.06	2.10 ± 0.03^§^
0.1	1.34 ± 0.05^#^	1.76 ± 0.26	1.30 ± 0.08^#^	1.64 ± 0.07^#^
0.5	1.34 ± 0.11^#^	1.74 ± 0.07	1.22 ± 0.17^#^	1.89 ± 0.01^#^
1.0	1.09 ± 0.05^#^	1.98 ± 0.29	1.23 ± 0.06^#^	2.42 ± 0.32
2.5	1.50 ± 0.17	1.78 ± 0.23	1.32 ± 0.12^#^	2.24 ± 0.39
5.0	1.28 ± 0.05^#^	2.16 ± 0.20*	1.33 ± 0.07^#^	2.27 ± 0.24
10.0	1.25 ± 0.03^#^	2.39 ± 0.32	0.84 ± 0.09^#^	2.57 ± 0.31
20.0	1.36 ± 0.05^#^	2.31 ± 0.33*	1.32 ± 0.06^#^	2.54 ± 0.04**
Statistical significance	^#^PPS < control (*P *< 0.05)	*PPS > control (*P *< 0.05)	^#^PPS < control (*P *< 0.05)	^§^Matrix > media (*P *< 0.05), ^#^PPS < control (*P *< 0.05), **PPS > control (*P *< 0.005)

Concentration effects of pentosan polysulfate (PPS) on hyaluronan (HA) deposition in the media and matrix of 7-day and 10-day micromass cultures of human mesenchymal progenitor cells (MPCs). Data are mean ± standard deviation of triplicate culture values.

### Real-time quantitative PCR

**Figure 7:**
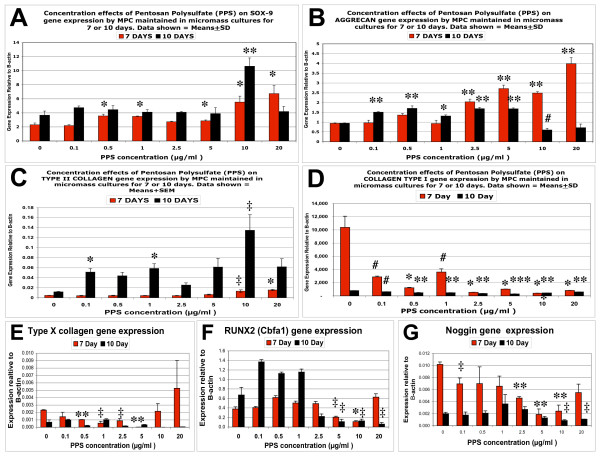
**Effects of pentosan polysulfate on gene expression by mesenchymal precursor stem cells in micromass cultures**. Bar graphs showing the concentration effects of pentosan polysulfate (PPS) on (a) SOX-9, (b) Aggrecan, (c) collagen type II, (d) collagen type I, (e) collagen type X, (f) RUNX2 (Cbfa1) and (g) Noggin gene expression by mesenchymal precursor stem cells (MPCs) in micromass cultures for 7 and 10 days as determined by real-time quantitative PCR. n = 3 for each PPS concentration. Data expressed as mean ± standard deviation for each PPS concentration after normalising for the expression of the housekeeping gene, β-actin. ^#^*P *< 0.05, *P < 0.01, **P < 0.001, ***P < 0.0001 gene expression relative to control.

For the most part, SOX-9 gene expression by MPCs was observed to be significantly upregulated by PPS at concentrations ≥ 0.05 μg/ml (*P *< 0.01) in 7-day MMC cultures (Figure 7a). In 10-day MMC, a strong upregulation of SOX-9 gene expression was observed at 10 μg/ml (*P *< 0.001), but this effect declined to basal levels at 20 μg/ml PPS (Figure 7a). Aggrecan gene expression in day-7 MMC followed a similar pattern to that observed for SOX-9, with the highest expression relative to control MMC occurring at 20 μg/ml PPS (*P *< 0.001) (Figure 7b). In the 10-day MPC cultures, however, upregulation of the aggrecan gene occurred over the PPS concentration range of 0.1 to 5.0 μg/ml (*P *< 0.01 to 0.001) but decreased at 10 and 20 μg/ml (Figure 7b). Collagen type II expression in MPCs was low and found to be only increased in the presence of 10 and 20 μg/ml PPS in 7-day cultures (*P *< 0.05) but was elevated at lower concentrations (0.1 and 1.0 μg/ml PPS) (*P *< 0.01) in the 10-day cultures (Figure 7c). Although collagen type I expression was considerably higher than collagen type II in all MPC cultures, it declined significantly in the 7-day and 10-day MMC containing PPS at all concentrations examined (*P *< 0.05 to 0.001) (Figure 7d).

The gene marker of chondrocyte hypertrophy, collagen type X, was suppressed in the 10-day MMC by PPS at concentrations of 0.1, 0.5 and 2.5 μg/ml (*P *< 0.05 to 0.001) (Figure 7e), while the osteogenesis marker gene, RUNX2, required the higher concentrations of 2.5 to 10.0 μg/ml to achieve significant downregulation (*P *< 0.05) (Figure 7f). Noggin expression was suppressed by PPS in 7-day cultures at 0.1 μg/ml (*P *< 0.05) and in 10-day cultures over the concentration range of 2.5 to 10 μg/ml PPS (*P *< 0.05 to 0.001) (Figure 7g).

### Effects of PPS on the differentiation of human MPCs

**Figure 8:**
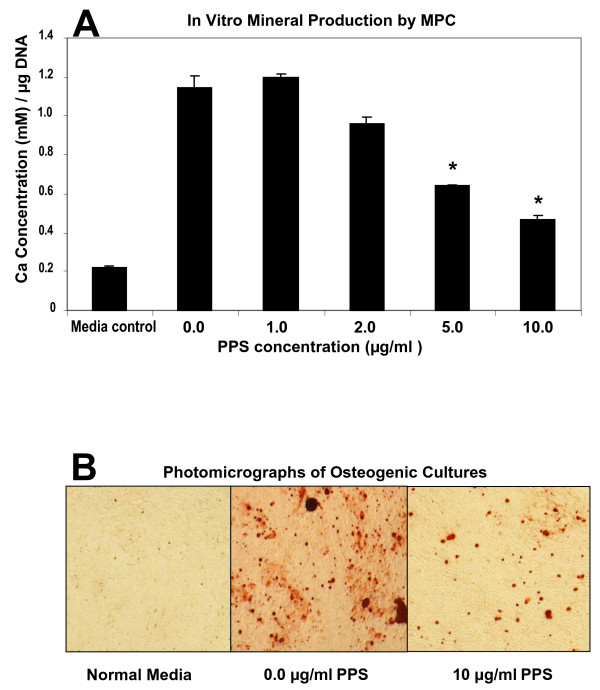
**Effects of pentosan polysulfate on human mesenchymal precursor stem cell differentiation: mineralisation assay**. Primary mesenchymal precursor stem cells (MPCs) were cultured in non-osteoinductive growth media (media control) or in osteoinductive conditions in the absence or presence of pentosan polysulfate (PPS) at the indicated concentrations. n = 3 for each PPS concentration studied. **(a) **Concentration of acid-solubilised calcium per well determined relative to the total amount of DNA per well. Data expressed as mean ± standard error of the mean for day 28. **(b) **Phase-contrast photomicrographs of cultures showing absence of mineralisation in the co-cultures with PPS (× 20). A statistically significant decrease (*P *< 0.01) in mineralised matrix formation was observed for PPS concentrations of 5 and 10 μg/ml. *P < 0.01 relative to controls.

**Figure 9:**
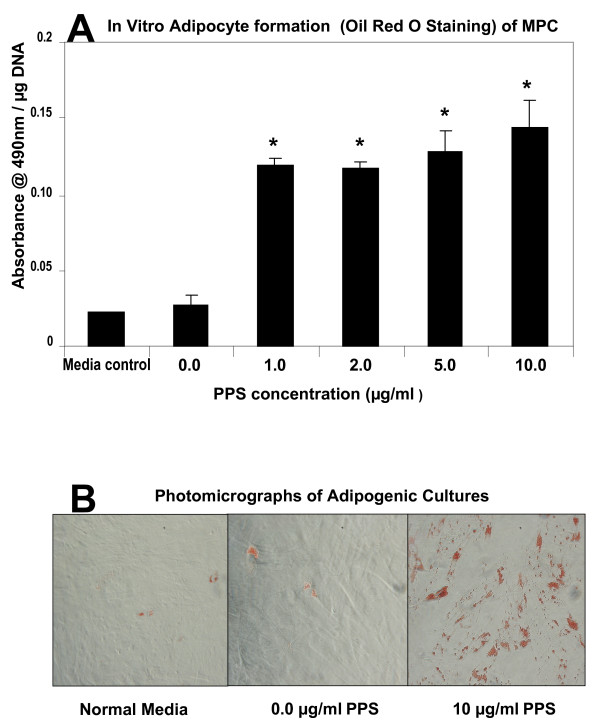
**Effects of pentosan polysulfate on human mesenchymal precursor stem cell differentiation: adipocyte formation**. Bar graph showing the concentration effects of pentosan polysulfate (PPS) on human mesenchymal precursor stem cell (MPC) adipogenic differentiation when cultured for 28 days in an adipogenic inductive medium. Primary MPCs were cultured in nonadipogenic growth media (media control) or under adipogenic conditions in the presence of PPS at the indicated concentrations. n = 3 for each PPS concentration studied. On day 28: **(a) **levels of lipid determined relative to the total amount of DNA per well, and **(b) **phase-contrast photomicrography of Oil Red O-labelled adipocytes (× 20). Data expressed as mean ± standard error of the mean. A statistically significant increase (*P *< 0.01) in adipocyte number was observed at PPS concentrations > 1.0 μg/ml. *P < 0.01.

Previous studies have shown that the STRO-3-positive human bone marrow-derived MPCs used in these studies undergo differentiation into osteocytes or adipocytes when cultured with the appropriate inductive media [9,13]. In the present study, however, co-culturing MPCs with 5 or 10 μg/ml PPS in an osteogenic media for 28 days suppressed their differentiation into the osteocyte/osteoblast lineage (*P *< 0.01) (Figure 8). In the presence of the adipogenic medium, PPS was found to enhance adipogenic differentiation of MPCs. This effect was significant at all PPS concentrations examined (*P *< 0.01) (Figure 9).

## Discussion

The present studies have demonstrated that PPS induces *in vitro *proliferation and chondrogenic differentiation of MPCs in a concentration-dependent and time-dependent manner. These activities were manifest over a PPS concentration range of 1 to 20 μg/ml, with 2.5 and 5.0 μg/ml showing the strongest effects. At the higher concentration of 20 μg/ml PPS, however, in 7-day cultures a possible decline in MPC chondrogenesis was signalled by the upregulation of collagen type X gene expression and decreased expression of RUNX2, but these variations were not statistically significant. Similar dose-dependent changes in chondrogenic differentiation have been reported with glucosamine when cultured with MSCs in the presence of TGF-β [34]. The enhanced production of the cartilage-specific phenotypic markers, PGs and type II collagen, by MPCs in the presence of PPS was demonstrated in MMC using a quantitative radiochemical assay and semiquantitatively by immunostaining with a type II collagen antibody. These assays were supported qualitatively by the positive staining of the MMC for the presence of PGs using Alcian blue dye and in histological sections of pellet cultures by Toluidine blue dye and type II collagen antibody staining. Significantly, immunostaining of serial sections for collagen type I clearly demonstrated diminished levels of this protein in pellets grown in the presence of PPS.

The aforementioned findings were consistent with the real-time PCR results, which showed the corresponding upregulation of SOX-9, aggrecan and type II collagen gene expression but downregulation of type I collagen expression by MPCs cultured in the presence of PPS. In addition, MPC expression of gene markers for chondrocyte hypertrophy and osteogenesis, type X collagen and the key transcription factor for bone formation, RUNX2, were markedly downregulated by PPS at concentrations that induced the strongest MPC chondrogenic differentiation. Furthermore, the experiments designed to promote MPC osteogenic differentiation using osteogenic-inductive culture media failed to achieve this effect in the presence of 5 and 10 μg/ml PPS, although adipogenic differentiation was increased in the adipogenic culture medium.

The establishment of MSCs in pellet cultures or MMS has been shown to favour their condensation, proliferation and differentiation along the chondrocytic lineage [6,7,28-30,35]. In the absence of media supplemented with growth factors such as TGF-β3, BMPs, fibroblast growth factor, insulin-like growth factor (IGF) or insulin, however, MSCs normally required several weeks to exhibit significant chondrogenic differentiation. Moreover, such cultures generally progressed towards a mixed cartilage phenotype with the upregulation of type I collagen and type X collagen [35]. In this context, it should be noted that the chondrogenic differentiation of MPCs mediated by PPS was demonstrated without the addition of growth factors or other chondroinductive supplements and failed to upregulate type I collagen and collagen type X expression. Furthermore, our unpublished studies using MPCs grown in collagen sponges or murine MSCs in MMC or pellet cultures have shown that PPS acts synergistically with insulin or TGF-β3 in promoting chondrogenic differentiation of these cells.

To our knowledge this is the first study to show that adult MPCs in high-density MMC synthesised HA. In the 7-day control cultures HA was localised equally between the media and extracellular matrix, but in the 10-day cultures more HA was deposited in the extracellular matrix. MPCs cultured in the presence of PPS showed increased deposition of HA in the matrix while levels in the media declined proportionally. A possible explanation for this shift in distribution could relate to the interaction of HA with newly synthesised PG monomers from the differentiating MPCs to form the PG aggregates characteristic of hyaline cartilage. Enhanced binding of HA to pericellular hyaladherins and MPC receptors (for example, CD44) upregulated by PPS, however, could also be a consideration.

Using other related polysaccharides in the PG biosynthesis assay provides evidence for the specificity of PPS as a promoter of MSC chondrogenesis. In 5-day MPC MMC in which HA was substituted for PPS, stimulation of PG synthesis occurred at 1 μg/ml but suppression was observed at concentrations > 5 μg/ml. DS, which has a similar charge density and molecular weight to PPS, strongly inhibited PG synthesis by MPC at all concentrations > 1 μg/ml, while heparin showed no effect over the concentration range used.

Native HA is known to have negative effects on chondrogenesis during limb morphogenesis [36] but there are limited data available on its effects on adult MSCs. In alginate bead cultures of MSCs, incorporation of high molecular weight HA into the beads enhanced chondrogenic differentiation at concentrations of 50 to 1,000 μg/ml, but the effects at lower concentrations were not reported [37]. MSCs grown in pellet cultures exposed to HA of varying molecular weight for up to 21 days were reported to have little or a negative effect on chondrogenesis [38]. The experimental outcome was also independent of the HA molecular weight used [38]. While we are unaware of any previous reports describing the ability of DS to influence chondrogenesis in adult MPC cultures, heparin, dermatan sulfate, DS and heparan sulfate have been reported to stimulate nodule formation and growth in 23/24-day chick mesenchymal MMS [39]. In another report using the same experimental culture system, however, heparin and HA were found to inhibit chondrogenesis [40].

Heparan sulfate PGs have been shown by several groups to play a significant role in the control and differentiation of MSCs. Indeed, heparan sulfate PGs have been nominated to be the master regulators of MSC proliferation and differentiation in the bone marrow niche as well as during tissue morphogenesis, neovascularisation and wound repair [41]. Many of the heparan sulfate PGs are present on cell surfaces as receptors where they bind growth factors and orchestrate their delivery and signal transduction to the cell nucleus [41]. These processes have been most extensively studied using the fibroblast growth factors [41]. BMPs are well documented as mediators of MSC differentiation [14,41] but were not monitored in the present studies. However, expression of Noggin, an antagonist of BMPs [42,43], was significantly downregulated by PPS over the concentration range of 1 to 20 μg/ml. The effects of PPS on TGF-β protein levels or expression were not examined in this study but together with other important intracellular signalling proteins are presently under investigation.

PPS, heparin and heparan sulfate share common structural features in that they all contain sulfate ester group substituents in multiple ring positions of linear polysaccharides. In PPS, the sulfate esters are symmetrically located in the two and three positions of every pentose ring. Heparin and heparan sulfate also contain a sulfate ester group in the two position but the other sulfate is located on the amino group of the same glucosamine ring. Unlike PPS, heparin and heparan sulfate are heterogeneous molecules and can have additional sulfate substitution in the iduronic acid ring as well as a number of other structural variations. Nevertheless, the 2/3 sulfate spatial pattern, which is common to all these sulfated polysaccharides, has been used to rationalise their cross-reactivity with certain monoclonal antibodies [18], their inhibitory activity against granulocyte elastase and insulin-like growth factor binding protein (IGFBP)-5 proteolysis and certain serine proteases of the complement, haemostatic and fibrinogenic systems [18,19,44,45]. It was therefore surprising to find in the present studies that neither heparin nor DS promoted MPC chondrogenesis, at least as assessed by the biosynthesis of PGs, indicating that the presence of the highly negatively charged sulfate groups in the two and three positions of polysaccharide chains were not critical structural determinants of chondrogenic differentiation by MPCs.

In the present real-time PCR experiments relative to β-actin, collagen type I was the most strongly expressed gene, followed by IGFBP-3. PPS at 1.0 and 10 μg/ml significantly increased MPC expression of IGFBP-3; however, IGFBP-5 was only weakly expressed and marginally upregulated by PPS (data not shown). IGFBP-3 has a strong affinity for IGF-1 and, like IGFBP-5, can modulate its bioavailability and receptor binding - but in addition IGFBP-3 exhibits IGF-1-independent effects on MSCs [4,46]. These direct effects of IGFBP-3 on MSCs include suppression of proliferation, chondrogenesis, adipogenesis and promotion of apoptosis [4,46].

Both IGFBP-3 and IGFBP-5 are degraded by the disintegrin metalloprotease, ADAM-12 (meltrin-α) [47,48], which therefore has the potential to influence the physiological activities of these binding proteins. Importantly, ADAM12 has been reported be highly expressed by the chondrocytes of osteoarthritis cartilage [49], a sizable proportion of which may be MPCs [50]. Moreover, the extent of expression of ADAM12 by chondrocytes correlated with pathological markers of disease progression such as cell cloning and proliferation [49].

Heparin and heparan sulfate have recently been shown to bind to ADAM-12s and inhibit its proteolytic activity [51]. In contrast, PPS enhanced ADAM-12s degradation of IGFBP-3 [51]. This finding coupled with our observations that PPS promoted MPC proliferation, chondrogenesis, adipogenic differentiation while decreasing osteogenesis, and apoptosis leads us to suggest that some of these events could be mediated by the increased proteolysis of IGFBP-3 by ADAM-12 due to its interaction with PPS. Furthermore, enhanced proteolysis of IGFBP-3 by ADAM12 would increase the bioavailability of IGF-1, thereby also supporting MPC proliferation and chondrogenic differentiation. On the other hand, it has also been shown that the apoptosis-potentiating and anti-proliferative activity of IGFBP-3 on MSCs may be antagonised through a TGF-β-dependent ERK pathway [52], suggesting that the proliferative, chondrogenic and anti-apoptotic effects of PPS on MPCs could be mediated by their increased production of TGF-β.

While additional investigations will be required to clarify the mechanism of action of PPS on MPCs in more detail, the outcomes of the present studies have confirmed our working hypothesis that this drug could be used in place of TGF-β or other related growth factors to induce chondrogenic differentiation of these multipotent cells.

## Conclusions

To our knowledge this is the first study to demonstrate that the anti-osteoarthritis drug PPS possesses the ability to promote MPC proliferation and chondrogenic differentiation while suppressing osteogenic expression and bone formation. Although additional studies are still required to establish that these observed effects are also mediated *in vivo*, in principal the combination of PPS with MPCs alone or when integrated into a bioscaffold could offer new therapeutic opportunities for the repair and reconstitution of injured and degenerate cartilaginous tissues of the musculoskeletal system.

## Abbreviations

ADAM: a disintegrin and metalloprotease (adamlysin); ADAMTS: adamalysin with thrombospondin motifs; BMP: bone morphogenetic protein; DS: dextran polysulfate; DMEM: Dulbecco's modified Eagle's medium; ELISA: enzyme-linked immunosorbent assay; FBS: foetal bovine serum; HA: hyaluronan; IGF: insulin-like growth factor; IGFBP: insulin-like growth factor binding protein; IL: interleukin; IFN: interferon; MMC: micromass cultures; MPC: mesenchymal precursor stem cell; MMP: matrix metalloproteinase (collagenase); MSC: mesenchymal stem or stromal cell; PBS: phosphate-buffered saline; PCR: polymerase chain reaction; PPS: pentosan polysulfate; PG: proteoglycan; S-GAG: sulfated glycosaminoglycan; TGF: transforming growth factor; WST-1: 4-(3-(4-iodophenyl)-2-(4-nitrophenyl)-2H-5-tetrazolio)-1,3-benzene disulfonate.

## Competing interests

PG, ACWZ and SG are paid consultants for Mesoblast Ltd, who provided the MPCs used for this study. SI is a Director and employee of Mesoblast, who did not finance this manuscript. JW and SS are employees of Proteobioactives Pty Ltd. PG holds shares in Proteobioactives Pty Ltd but did not receive fees, funding or a salary from this company. PG, ACWZ, SG and SI are Mesoblast Ltd shareholders. PG has filed a PCT application related to the content of the manuscript but did not receive reimbursements, fees, funding, or salary from an organisation that holds or has applied for patents relating to the content of the manuscript.

## Authors' contributions

PG was responsible for the initial study concept as well as the experimental design, execution and interpretation of the research. He was also responsible for the drafting of the manuscript. JW and SS undertook all of the MPC culture experiments, ELISAs, and immunohistochemical, biochemical and radiochemical assays conducted at Proteobioactives Pty Ltd, Sydney. ACWZ and SG undertook the WST-1 proliferation, apoptosis, osteogenic, adipogenic and real-time PCR assays at the Hanson Institute, Adelaide. ACWZ, SG and SI all contributed to the interpretation of the experimental results and drafting of the final manuscript. All authors read and approved the submitted manuscript.
